# A sorghum NAC gene is associated with variation in biomass properties and yield potential

**DOI:** 10.1002/pld3.70

**Published:** 2018-07-23

**Authors:** Jingnu Xia, Yunjun Zhao, Payne Burks, Markus Pauly, Patrick J. Brown

**Affiliations:** ^1^ Department of Crop Sciences University of Illinois at Urbana Champaign Urbana Illinois; ^2^ Department of Plant and Microbial Biology University of California, Berkeley Berkeley California; ^3^ Present address: Department of Biochemistry University of Oxford Oxford UK; ^4^ Present address: Brookhaven National Lab Upton New York; ^5^ Present address: Chromatin Inc. Lubbock Texas; ^6^ Present address: Heinrich‐Heine University Duesseldorf Germany; ^7^ Present address: University of California, Davis Davis California

**Keywords:** biomass composition, biomass moisture, grain‐filling, secondary cell wall

## Abstract

*Sorghum bicolor* is a C_4_ grass widely cultivated for grain, forage, sugar, and biomass. The sorghum *Dry Stalk (D)* locus controls a qualitative difference between juicy green *(dd)* and dry white *(D‐)* stalks and midribs, and co‐localizes with a quantitative trait locus for sugar yield. Here, we apply fine‐mapping and genome‐wide association study (GWAS) to identify a candidate gene underlying *D*, and use nearly isogenic lines (NILs) to characterize the transcriptional, compositional, and agronomic effects of variation at the *D* locus. The *D* locus was fine‐mapped to a 36 kb interval containing four genes. One of these genes is a NAC transcription factor that contains a stop codon in the NAC domain in the recessive *(dd)* parent. Allelic variation at *D* affects grain yield, sugar yield, and biomass composition in NILs. Green midrib (*dd)* NILs show reductions in lignin in stalk tissue and produce higher sugar and grain yields under well‐watered field conditions. Increased yield potential in *dd* NILs is associated with increased stalk mass and moisture, higher biomass digestibility, and an extended period of grain filling. Transcriptome profiling of midrib tissue at the 4–6 leaf stages, when NILs first become phenotypically distinct, reveals that *dd* NILs have increased expression of a miniature zinc finger (MIF) gene. MIF genes dimerize with and suppress zinc finger homeodomain (ZF‐HD) transcription factors, and a ZF‐HD gene is associated with midrib color variation in a GWAS analysis across 1,694 diverse sorghum inbreds. A premature stop codon in a NAC gene is the most likely candidate polymorphism underlying the sorghum *D* locus. More detailed understanding of the sorghum *D* locus could help improve agronomic potential in cereals.

## 1 INTRODUCTION

*Sorghum bicolor*, a versatile and resilient C_4_ grass, is an important staple cereal in semiarid areas of Africa and Asia (Paterson et al., 2009). In addition to its use as a cereal, sorghum is widely grown for production of forage, ethanol, heat, and electricity (Rooney, Blumenthal, Bean, & Mullet, 2007). Reflecting this diversity of end uses, sorghum varieties vary greatly in plant height, flowering time, grain yield and harvest index, and sorghum vegetative biomass varies greatly in sugar content, composition, digestibility, and mechanical strength (Mullet et al., 2014; Stefaniak et al., 2012). Despite the identification of several major loci affecting sorghum plant height (Multani et al., 2003; Yamaguchi et al., 2016) and flowering time (Murphy et al., 2011; Murphy et al., 2014; Yang, n.d), no major loci affecting vegetative biomass properties in sorghum have previously been identified.

Vegetative biomass properties are largely determined by the architecture of the vascular system, xylem, and phloem, which provide mechanical support, allow water and nutrient acquisition, and mediate transport of photoassimilates and signaling molecules from source to sink tissues (Bihmidine, Hunter, Johns, Koch, & Braun, 2013). Grass leaves and inflorescences are major source and sink organs, and the genetic control of their architecture has been intensely studied (Kellogg, 2007; Pautler, Tanaka, Hirano, & Jackson, 2013; Tanaka, Pautler, Jackson, & Hirano, 2013). However, the architecture of the vascular system also plays a crucial role in determining cereal grain yields. During grain filling, continued acquisition of water, sugars, and nitrogen is accompanied by re‐mobilization of transient storage reserves, and stalk strength must be maintained throughout senescence to protect the drying grain from lodging (Peiffer et al., 2013). The genetic control of vascular system architecture has been studied primarily in model systems (Handakumbura & Hazen, 2012), where genetic effects on agronomic performance may not be easily detected.

The large family of NAC transcription factors (TFs), named after founding members NAM, ATAF, and CUC (Fang, You, Xie, Xie, & Xiong, 2008), plays a critical role in plant vascular development (Ko, Jeon, Kim, Kim, & Han, 2014; Nakano, Yamaguchi, Endo, Rejab, & Ohtani, 2015). A subset known as the secondary wall NACs (SWNs) act as master regulators of secondary cell wall development in vascular tissues (Handakumbura & Hazen, 2012; Zhong, Demura, & Ye, 2006). Conservation of SWN function in moss hydroids and stereids, which, respectively, conduct water and lend mechanical support to the moss gametophyte, provides evidence that these structures are homologous to sporophytic vascular systems in higher plants (Xu et al., 2014). SWNs activate a subset of MYB transcription factors that contain secondary wall NAC binding elements (SNBEs) in their promoters, inducing secondary cell wall thickening and deposition of cellulose, hemicellulose and lignin (Zhong, Lee, & Ye, 2010). NAC TFs also act downstream of MYB TFs. During sieve element maturation, for example, the *Altered Phloem Development (APL)* gene both drives the expression of NAC045 and NAC086 and is itself regulated by NAC020 (Bonke, Thitamadee, Mahonen, Hauser, & Helariutta, 2003; Furuta et al., 2014). The extreme complexity of the secondary cell wall gene regulatory network in Arabidopsis is driven by functional redundancy, feed‐back and feed‐forward loops, and combinatorial control enabling functional fine‐tuning (Taylor‐Teeples et al., 2014).

The *Dry Stalk (D)* locus in sorghum conditions a difference between dry, pithy white stems and midribs *(D−)*, and juicy green stems and midribs *(dd)* (Smith & Frederiksen, 2000). A previous genome‐wide association study (GWAS) in sweet sorghum mapped large‐effect QTL for sugar yield, juice volume, and stalk moisture to the *D* locus (Burks, Kaiser, Hawkins, & Brown, 2015). In this study, we fine‐map the *D* locus to a 36 kb region, identify a premature stop codon in a NAC transcription factor as a candidate polymorphism, and show that nearly isogenic lines (NILs) segregating for the premature stop codon differ significantly in sugar yield, grain yield, and biomass composition.

## 2 METHODS

### 2.1 Plant material and controlled growth conditions

A dominant white midrib allele *(D)* was introduced into a genetic male sterile *(ms3/ms3)* version of Tx623, which carries a recessive green midrib allele *(d),* through four generations of backcrossing. Homozygous *D/D‐Ms3/Ms3* and *d/d‐Ms3/Ms3* seed stocks were derived from a single BC_4_ plant by selfing. Three replications of greenhouse plantings were performed on 1/7/16, 2/16/16, and 3/16/16, planted 2 seeds per cell in 6 by 8 flats and thinning to 1 plant per cell at 7 days after planting. Midrib color changes were recorded using a MiScope (Zarbeco, USA) at V4, V6, and V8, and midrib tissue for RNAseq was sampled at V4 and V6 into liquid N.

### 2.2 Field experiments

For sugar yield measurements, paired rows of NILs were planted in six replicates at a single location in Urbana, IL in summer 2014, and phenotyped as previously described (Burks et al., 2015). For grain and stalk dry matter and moisture measurements, paired 2‐row plots of NILs were planted in four replicates at each of two locations (Urbana and Savoy, IL), and samples were pooled from 2‐6 individual representative plants per row for each time point. Immature grain weight was estimated by clipping individual panicle branches from the rachis, stalk weight was measured after stripping off leaf blades, and moisture was estimated by weighing samples before and after oven‐drying at 45°C for 5 days. Midrib color in the GWAS panel was phenotyped as a binary trait (green vs white, Supporting information Table S7) in the youngest leaf at ~45 days after planting. All field experiments were machine planted in rows 10′ long with 30″ row spacing. Seeds were treated with Apron fungicide (Syngenta, USA) and Concep II seed safener (Syngenta, USA) before planting, and weeds were controlled through use of a pre‐emergent herbicide (Bicep). All raw agronomic data (Supporting information Tables S5 and S8) and raw compositional data (Supporting information Table S9) were analyzed by ANOVA in R using a linear model with location, block nested within location, and NIL genotype as fixed effects. We present *p*‐values for genotype effects, and boxplots show residuals from a model including location and block effects.

### 2.3 Fine‐mapping and GWAS

Two MITE‐based indel markers (Supporting information Table S10) were used to screen 1,132 BC_3_ and BC_2_F_2_ individuals. Subsequent genotyping‐by‐sequencing was performed in both putative recombinants and a GWAS panel of 1,624 diverse sorghum accessions using the two‐enzyme GBS protocol with PstI‐HF and HinP1I enzymes (Poland, Brown, Sorrells, & Jannink, 2012), followed by alignment to v3 of the reference genome (www.phytozome.org) using bowtie2 (Langmead & Salzberg, 2012) and SNP calling using the TASSEL5 GBSv2 pipeline (Glaubitz et al., 2014). Imputation of biparental recombinants was performed using FSFHap (Swarts et al., 2014) in TASSEL, and imputation of the GWAS panel was performed using Beagle4 (Browning & Browning, 2007). Midrib color GWAS was performed using the mixed linear model implemented in GAPIT, using model selection to choose the optimal number of principal components, which was zero. A minor allele frequency cutoff of 5% resulted in 50,899 SNPs for testing, and only associations significant at a false discovery rate of less than 5% (*q* < 0.05) are reported. GBS data for this project have been deposited at the Illinois Data Bank (https://databank.illinois.edu/).

### 2.4 Lignocellulosic compositional analysis

Lyophilized tissue samples were ground to a fine powder using three 5 mm metal balls in 2 ml plastic tubes (Retsch Ball mill, 2 times at 25 Hz for 2.5 min) and washed sequentially with 70% ethanol, 1:1 (v:v) methanol:chloroform, and acetone. The alcohol‐insoluble cell wall material was further destarched with alpha amylase (Sigma) and Pullulanase M2 (Megazyme) in 0.1 M Citrate buffer pH 5.0. The destarched material was aliquoted for different compositional assays. 1 mg of destarched cell wall was hydrolyzed in 2 M Tri‐fluoroacetic acid (TFA) heated to 121°C for 90 min followed by a stream of dried air using nitrogen. Dried samples were re‐suspended in water, centrifuged, and the supernatant was collected for monosaccharides analysis and the pellet was dried for crystalline cellulose measurements. TFA‐soluble samples were analyzed using High Performance Anion Exchange liquid Chromatography with Pulse Amperometric Detection (HPAEC‐PAD) according to de Souza, Hull, Gille and Pauly (2014). Neutral sugars were separated via a CarboPac PA20 column, while a CarboPac PA200 was used to separate uronic acids. Three distinct programs were used to resolve the sugars of interest. Samples were run at a flow rate of 0.4 ml/min and gradients consisted of (a) 2 mM NaOH for 20 min followed by a 5 min 100 mM flush and subsequent 5 min at 2 mM (neutral sugar separation 1; excludes xylose and mannose); (b) 18 mM NaOH for 15 min followed by a 5 min 100 mM flush and subsequent 7 min at 18 mM (neutral sugar separation 2; excludes rhamnose and arabinose); (c) 0.1 M NaOH with a gradient of 50–200 mM sodium acetate from 0 to 10 min followed by a 2 min 200 mM sodium acetate flush returning to 50 mM for 2.9 min (uronic acid separation). Crystalline cellulose was measured using the Updegraf method and the released glucose was measured using the anthrone assay (Updegraff, 1969; Laurentin & Edwards, 2003). Lignin content and lignin composition were measured using the ultra‐violet acetyl bromide lignin method and a thioacidolysis procedure, respectively, according to Foster, Martin and Pauly (2010). Saccharification yield of cell wall materials was determined after enzymatic treatment. In brief, 1 mg of destached cell wall was incubated with 0.5 μl Accellerase 1,500 enzyme mix (Gencor) in 1 ml of 50 mM citrate buffer (pH 4.5), shaking at 250 rpm at 50°C for 20 hr. Solubilized glucose and xylose were detected on a Bio‐Rad HPX‐87H, 300 × 7.8 mm column in an Shimadzu UFLC chromatography system. The elution profile encompassed 0.01 N sulfuric acid in 15 min at 0.6 ml/min and column temperature is 50°C.

### 2.5 Phylogenetic tree construction and sequence alignment

*SbNAC074a* homologs from *Arabidopsis thaliana*,* Oryza sativa subsp. Japonica, Sorghum bicolor, Zea mays, Setaria italica,* and *Glycine max* were extracted from UNIPROT (http://www.uniprot.org). The full length sequence of SbNAC074a from the *DD* NIL, which doesn't have the null mutation, was BLASTED in UNIPROT (http://www.uniprot.org) and homologs from *Arabidopsis thaliana (sequence version 1), Oryza sativa subsp. Japonica (sequence version 2 for Os04 g43560, others were sequence version 1), Sorghum bicolor (sequence version 1), Zea mays (sequence version 1), Setaria italica (sequence version 1), and Glycine max (sequence version 1)* were identified from the BLAST result. Alignment was performed using MUSCLE in MEGA7 with default settings, and CLUSTALW2_Phylogeny was used with distance correction off, gaps excluded, and the UPGMA clustering method to create the tree. Visualization was performed using EVOLVIEW (http://www.evolgenius.info/evolview/#login).

### 2.6 Gene expression analysis

Total RNA was extracted from greenhouse‐grown midrib tissue of the youngest fully expanded leaf of *DD* and *dd* NILs at the four‐leaf and six‐leaf stages using Spectrum™ Plant Total RNA Kit (Sigma‐Aldrich, USA), following the manufacturer's protocol. All RNA samples were digested with DNase I (New England Biolabs, USA), and rtPCR was performed using M‐MuLV reverse transcriptase (New England Biolabs, USA). RNAseq libraries were prepared and sequenced at Roy J. Carver Biotechnology Center at the University of Illinois using single‐end, 100 bp reads on a HiSeq2500 instrument. Reads were processed using HISAT2 and StringTie, and the stattest function in the R package “Ballgown” was used to test for differential gene expression between *DD* and *dd* NILs using 3 biological replicates at each developmental stage, controlling the false discovery rate at 0.05. The R package “WGCNA” was used to construct co‐expression modules using the arguments power = 8, minModuleSize = 20, and mergeCutHeight = 0.05. RNAseq reads for this project have been deposited at the Illinois Data Bank (https://databank.illinois.edu/).

## 3 RESULTS

### 3.1 Creation and characterization of nearly isogenic lines for the *D* locus

**Figure 1:**
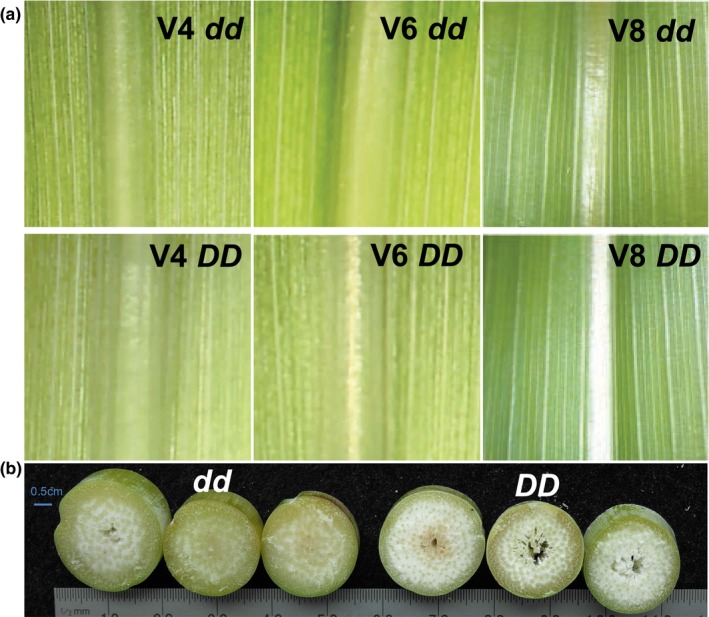
Phenotypic differences between *DD* and *dd* nearly‐isogenic lines (NILs). (a) Midribs of the youngest fully expanded leaf in *dd* and *DD* NILs at V4, V6, and V8 stages at 2, 3, and 4 weeks after planting, respectively. (b) Cross‐sections through the second stalk internode above ground level at anthesis (10 weeks after planting (WAP)), with 0.5 cm scale bar

A white midrib allele *(D)* was introduced into the green midrib Tx623 background *(dd)* through four generations of backcrossing, and homozygous *DD* and *dd* seed stocks were derived from a single BC_4_ plant by selfing. NILs appear identical until the sixth‐leaf stage, when a narrow band of pithy white tissue first appears in *DD* midribs, becoming wider and more distinct in successive leaves (Figure 1a). At anthesis, *DD* stalks are visibly drier and pithier than *dd* stalks (Figure 1b). All sampling times and tissues presented in this study are summarized in Supporting information Table S1.

### 3.2 GWAS and fine‐mapping

**Figure 2:**
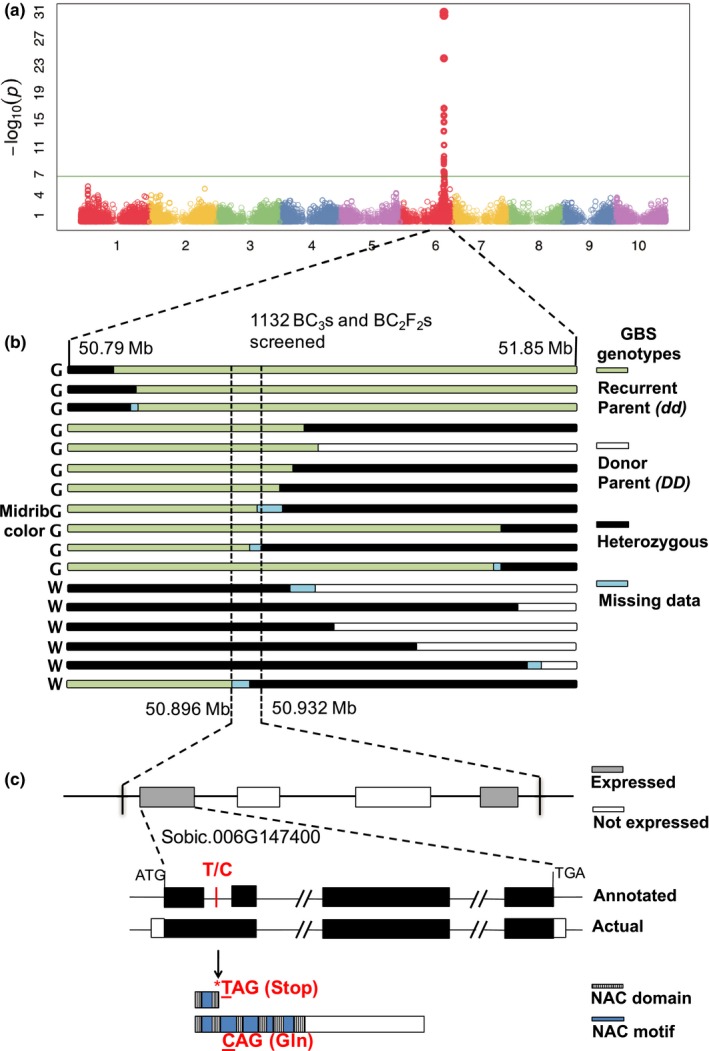
Genome‐wide association study (GWAS) and fine‐mapping of the sorghum *D* locus. (a) Midrib color GWAS in 1,624 sorghum inbreds, with a major peak at ~51 Mb on chr6. (b) Screening 1,132 individuals with flanking markers yielded 17 recombinants, which were GBSed to define a 36 kb interval. (c) Two of four genes in the 36 kb interval are expressed, one of which is a NAC transcription factor (Sobic.006G147400) with a stop codon in the first exon disrupting the NAC domain. This gene was annotated incorrectly in the sorghum reference genome (Tx623), which has the stop codon and a recessive *d* allele

Midrib color was scored as a binary trait (green/white) in the flag leaf at anthesis in a large panel (*n* = 1624) of sorghum inbreds, revealing a single very strong association at ~ 51 Mb on chromosome 6 (Figure 2a; Supporting information Table S2). Polymorphic markers flanking this region were used to screen 1132 BC_3_ and BC_2_F_2_ individuals. Putative recombinant individuals were subjected to genotyping‐by‐sequencing (GBS), confirming 17 recombinants and defining a ~36 kb interval that co‐segregates perfectly with the phenotype (Figure 2b, Supporting information Table S3). This interval contains four predicted genes, of which two are expressed in midrib tissue at either the fourth‐ or sixth‐leaf stage (Supporting information Table S4): a NAC transcription factor (Sobic.006G147400) and a threonine aldolase (Sobic.006G147600). The two most significant hits in the GWAS analysis are the two closest flanking SNPs to the NAC gene. Moreover, the NAC gene is the only gene in the interval with a different annotated exon‐intron structure compared to its closest homologs in other cereals, which is relevant because the sorghum reference genome is derived from Tx623, a recessive *dd* mutant. Reverse‐transcriptase PCR and cDNA sequencing confirms that the annotated first intron in Sobic.006G147400 does not exist, and that a T/C SNP in this region produces a stop codon in the *d* allele from Tx623 but not the contrasting D allele (Figure 2c). The premature stop codon in Tx623 eliminates most of the NAC domain.

### 3.3 Phylogeny and identification of homologs

**Figure 3:**
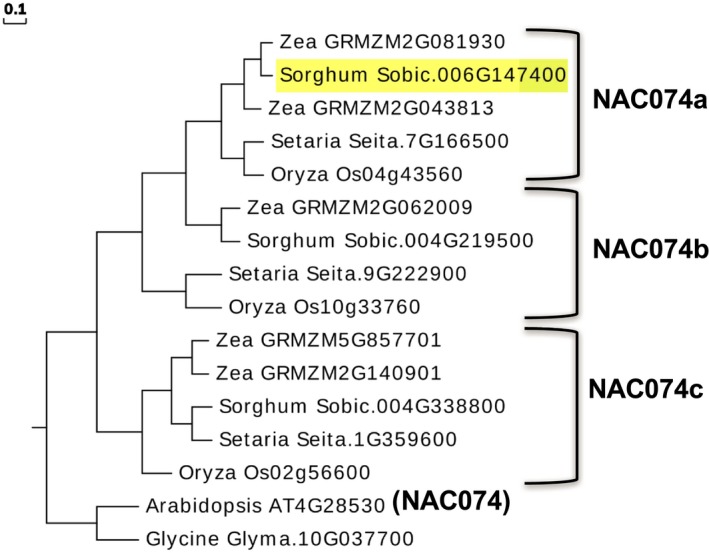
UPGMA tree of NAC074 genes in grasses. *SbNAC074a* is highlighted

The full‐length NAC protein, derived from an allele of Sobic.006G147400 lacking the premature stop codon, was used to search for homologous proteins in four grasses (*Oryza sativa subsp. japonica, Sorghum bicolor, Zea mays, Setaria italica)* and two dicots (*Arabidopsis thaliana* and *Glycine max*), which were aligned using MUSCLE in MEGA7 and used to construct a neighbor‐joining tree using UPGMA clustering (Figure 3). Syntenic regions on *Oryza* chromosome 4 and *Setaria* chromosome 7 each contain single orthologous copies of our sorghum NAC candidate, and co‐syntenic regions on *Zea* chromosomes 2 and 10 each contain intact homeologues derived from segmental duplication. These proteins are part of the NAC1 sub‐clade (Peng et al., 2015), and contain five conserved motifs in the N‐terminal NAC domains whereas the C‐termini are highly variable (Supporting information Figure S1). The closest *Arabidopsis* homolog is NAC074 (At4G28530), and we hereafter refer to the three co‐orthologous grass clades as NAC074a, NAC074b, and NAC074c, and to Sobic.006G147400 as *SbNAC074a*.

### 3.4 *dd* NILs show increases in stalk dry matter, soluble sugar yield, and grain yield

**Figure 4:**
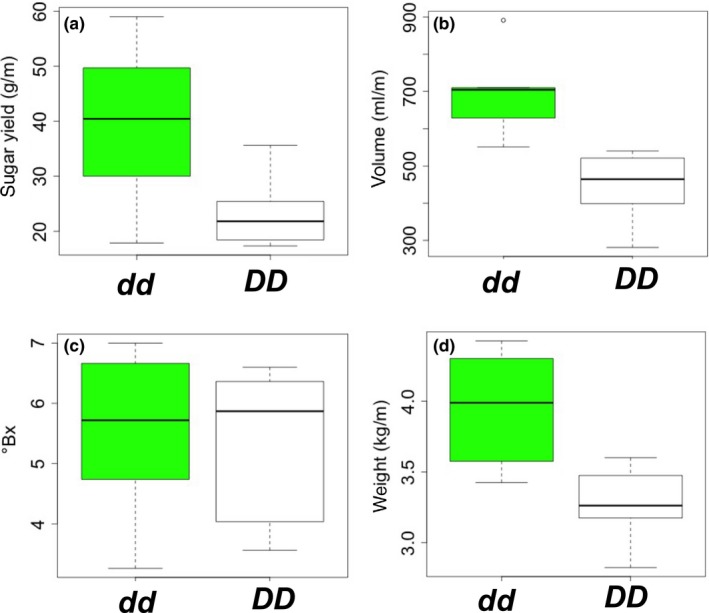
Sugar accumulation in *DD* and *dd* nearly‐isogenic lines (NILs). Total stalk tissue from 1 m of row was sampled at the hard dough stage, 4 weeks after anthesis. (a) Sugar yield (g/m); (b) Juice volume (ml/m); (c) Brix (g/ml); (d) Vegetative Wet Weight (kg/m). Sugar yield is calculated by multiplying juice volume and brix

**Figure 5:**
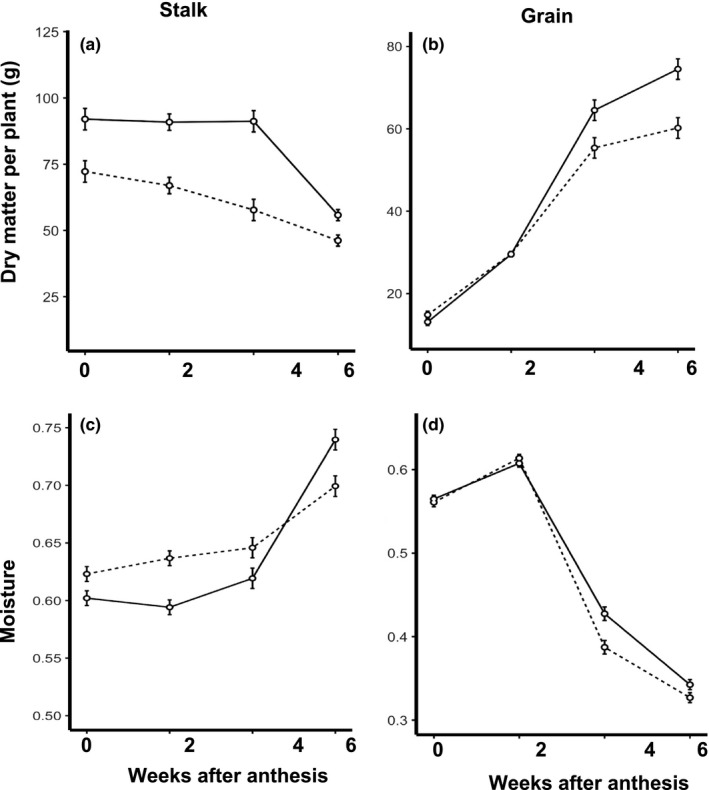
Grain filling of *DD* nearly‐isogenic lines (NILs) (dashed lines) and *dd* NILs (solid lines) under field conditions. Changes in dry matter (a, b) and moisture (c, d) were monitored at 2‐week intervals in stalk (a, c) and grain (b, d) from 0–6 weeks after anthesis

To confirm the previously‐reported association between the *D* locus and sugar yield, we grew NILs in six replications of paired rows, extracted total sugar from 1 meter (m) of row 30 days after anthesis (see Methods), and found that *dd* NILs yield nearly twice the sugar of *DD* NILs (Figure 4a, *p* < 0.001) despite being identical in plant height and flowering time (Supporting information Table S5). Sugar yield per meter of row (g/m; Figure 4a) is a function of juice volume (ml/m; Figure 4b) and the concentration of simple sugars in the juice (degrees Brix; Figure 4c), and we observe that increased yield in *dd* NILs is driven by increased juice volume, which in turn is associated with higher vegetative weight (kg/m; Figure 4d). Given the dramatic effect of the *D* locus on sugar yield, we next conducted a time series experiment to quantify its effects on the rate and extent of grain filling. Paired, two‐row plots of NILs were grown in four replicates at each of two locations, and dry matter and moisture of stalk and grain were monitored at 2‐week intervals beginning at anthesis. Overall, this grain filling period is characterized by movement of dry matter from the stalk to the grain, and by decreases in grain moisture relative to stalk moisture (Figure 5). Stalk dry matter is significantly higher in *dd* NILs at all stages, most notably at 4 weeks after anthesis when it is >58% higher (*p* = 0.004; Figure 5a). *dd* NILs also have higher grain moisture at 4 weeks after anthesis (*p* = 0.042; Figure 5d), and higher grain yield at 6 weeks after anthesis (*p* = 0.025; Figure 5b).

### 3.5 Compositional differences drive increased biomass digestibility of *dd* NILs

**Figure 6:**
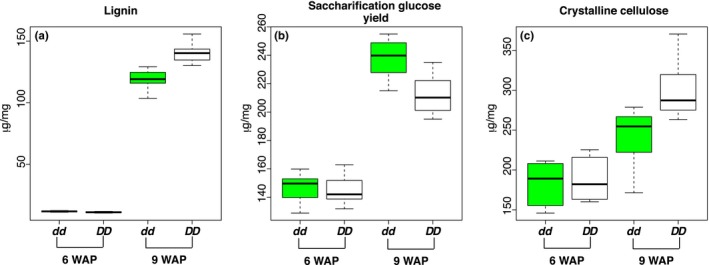
Compositional analysis of developing internodes in *DD* and *dd* nearly‐isogenic lines (NILs). (a) Lignin; (b) Saccharification glucose yield; (c) Crystalline cellulose. Tissues sampled included developing internodes at 6 weeks after planting (6 weeks after planting (WAP)) and the third internode below the inflorescence at 9 WAP

Nearly‐isogenic lines stalk tissue was subjected to detailed lignocellulosic compositional analysis at two growth stages: developing internodes from a 1 cm plug of tissue immediately below the shoot apical meristem at 6 weeks after planting (WAP), and the third internode below the inflorescence at 9 WAP, which coincided with the boot stage shortly before flowering. While tissues show relatively few compositional differences between NILs at 6 WAP, by 9 WAP *dd* NILs show reduced lignin (*p* = 0.007; Figure 6a) and increased glucose release following enzymatic saccharification (*p* = 0.049; Figure 6b), while their decrease in crystalline cellulose is not significant at *p* < 0.05 (*p* = 0.055; Figure 6c). *dd* NILs also show a lower ratio of syringyl to guaiacyl lignin monomers at 9 WAP (*p* = 0.029), and differences in acid‐hydrolyzed lignocellulosic monosaccharides at both stages (Supporting information Figure S2).

### 3.6 A miniature zinc finger (MIF) gene is upregulated in *dd* NILs

**Figure 7:**
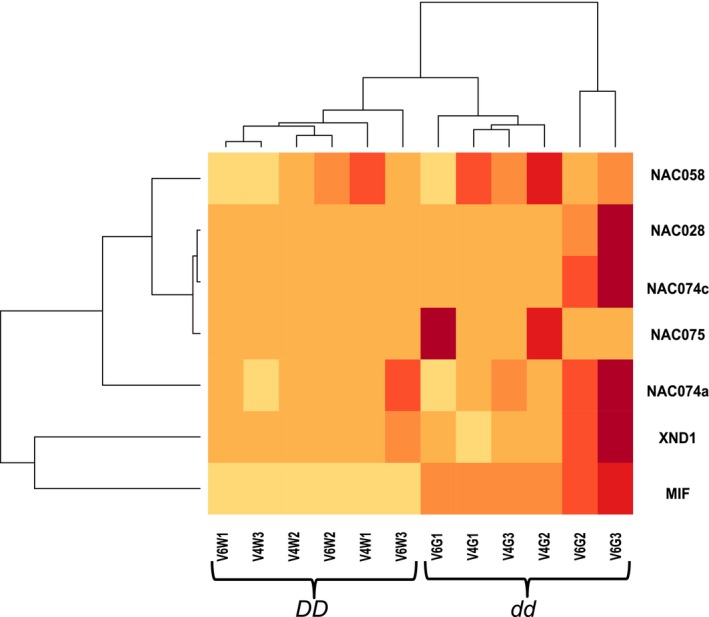
A Co‐expression of SbNAC074a, the differentially‐expressed miniature zinc finger (MIF) gene, and 5 other NAC transcription factors in V4 and V6 midribs of *DD* and *dd* nearly‐isogenic lines (NILs). Light and dark colors indicate low and high gene expression, respectively. Dendrograms reflect Euclidean distance and determine row and column order

Transcriptome profiling was performed on mRNA from NIL midrib tissue at the four‐leaf and six–leaf stages, just before and during the first appearance of phenotypic differences between the NILs (Figure 1a). Three independent biological replicates were obtained from pooled tissue from three separate greenhouse plantings, for a total of 12 samples across the two NILs and two stages. Co‐expression analysis using WGCNA's step by step network construction and module detection (Langfelder & Horvath, 2008) shows that *SbNAC074a* shares a regulatory module with 263 other annotated genes, including a group of five NAC transcription factors (Figure 7; Supporting information Table S6) that includes *SbNAC074c* but not *SbNAC074b* as well as two NACs associated with xylem development in Arabidopsis, *Xylem NAC Domain 1 (XND1)* and *NAC075* (Endo et al., 2015; Zhao, Avci, Grant, Haigler, & Beers, 2007). However, using the HiSAT‐Stringtie‐Ballgown pipeline (Pertea, Kim, Pertea, Leek, & Salzberg, 2016), only a single gene (Sobic.008G020700) is differentially expressed between DD and *dd* NILs (*q* < 0.01), showing no expression in *DD* NILs and mean expression of 21 and 35 FPKM in *dd* NIL midribs at the four‐leaf and six‐leaf stages, respectively. Sobic.008G020700 is annotated as a MIF gene, which are seed plant‐specific, truncated versions of ZF‐HD transcription factors (Hu & Ma, 2006) that dimerize with ZF‐HDs and suppress their transcriptional activation activity (Hong, Kim, Kim, Yang, & Park, 2011). Intriguingly, the only significant GWAS hit for midrib color other than our candidate NAC gene falls near a ZF‐HD transcription factor on chromosome 1 (Supporting information Table S2). However, this ZF‐HD transcription factor (Sobic.001G112500) shows no expression in any of the *DD* or *dd* samples.

## 4 DISCUSSION

In this study, we fine‐map the sorghum *D* locus to a four‐gene interval that includes two genes expressed in midrib tissue: a NAC transcription factor with a premature stop codon in the *dd* sorghum reference genome (Sobic.006G147400), and a threonine aldolase (Sobic.006G147600). Threonine aldolase controls the catabolism of threonine to glycine and acetaldehyde, and *Arabidopsis* homologs THA1 and THA2 are both expressed in vascular tissue. *tha1‐2* mutants show dramatic increases in seed threonine content, whereas *tha2‐1* mutants have a lethal albino seedling phenotype(Joshi, Laubengayer, Schauer, Fernie, & Jander, 2006). While we have not formally excluded the threonine aldolase as a candidate gene underlying the *D* locus in sorghum, our data from *dd* and *DD* NILs is more consistent with perturbation of NAC gene function. First, we note that the two most significant SNPs in our GWAS analysis are the two closest flanking markers to this NAC gene. Second, the most obvious mutant phenotype in *dd* NILs is the persistence of a green midrib throughout development. In *Arabidopsis,* a suite of NAC genes including ORE1, ANAC046, ANAC087, and ANAC100 positively regulate leaf de‐greening and senescence by binding directly to the promoters of chlorophyll catabolic genes (Oda‐Yamamizo et al., 2016, p. 46; Qiu et al., 2015). Therefore, we propose that loss of NAC gene function in *dd* NILs represses chlorophyll catabolism, causing the persistence of a green midrib. We have named this NAC gene *SbNAC074a*, after its closest homolog in Arabidopsis.

Previous study of genes orthologous to and co‐expressed with *SbNAC074a* suggest a role in xylem development. *Arabidopsis* NAC074 (At4G28530) is upregulated in xylem relative to phloem‐cambium and non‐vascular tissues (Zhao, 2005), and is one of many NAC genes upregulated during leaf senescence (Podzimska‐Sroka, O'Shea, Gregersen, & Skriver, 2015). The rice ortholog of *SbNAC074a* (Os04 g43560) is upregulated in panicle and root under drought stress in drought‐tolerant, but not drought‐susceptible, NIL backgrounds (Nuruzzaman et al., 2012). Here, we show that *SbNAC074a* is co‐expressed with other NAC transcription factors involved in xylem development, including homologs of Arabidopsis *XND1* and *NAC075*. *xnd1* mutants display a mild dwarfing phenotype associated with a reduction in tracheary element length, whereas overexpression of XND1 results in reduced formation of xylem vessels, expansion of the phloem, and increased starch storage in amyloplasts (Zhao et al., 2007).The authors suggest that XND1 may promote vessel elongation by repressing their terminal differentiation. Overexpression of NAC075 results in ectopic formation of xylem vessel elements (Endo et al., 2015) and rescues the pendent stem phenotype of *nst1‐nst3* double mutants, which results from complete loss of secondary cell wall deposition in xylem fibers.

Lignocellulosic compositional data are consistent with a role for *SbNAC074a* as a positive regulator of xylem development. *dd* NILs internodes have significantly reduced lignin. Lignin content has been positively correlated with xylem development and inversely correlated with lignocellulosic saccharification yields (Chen & Dixon, 2007), consistent with the observed increase in glucose yield following enzymatic saccharification in *dd* NILs. The increased digestibility of *dd* NILs comes with no obvious agronomic penalty in the dwarf grain sorghum background of Tx623, though we observe that green midrib accessions have higher stalk lodging than white midrib accessions in a biomass sorghum panel (Supporting information Figure S3). Strikingly, yields of soluble sugar, grain, and vegetative biomass are all significantly increased in *dd* NILs under well‐watered field conditions. These results are not easily explained and should be validated.

A premature stop codon in the NAC domain of SbNAC074a likely underlies allelic variation at the sorghum *Dry Stalk (D)* locus. NILs at the *D* locus show agronomic, compositional, and transcriptional differences. While *dd* NILs showed superior agronomic performance at our Illinois field sites with ample rainfall, the relative performance of *DD* and *dd* genotypes under terminal drought stress might differ. Better understanding of developmental perturbations mediated by the sorghum D locus could lead to enhancement of yield potential and climatic adaptation in cereals.

## COMPETING INTERESTS

The authors declare that they have no competing interests.

## AUTHORS’ CONTRIBUTIONS

JX, PB, and PJB analyzed and interpreted agronomic data. YZ and MP analyzed and interpreted compositional data. JX and PJB analyzed and interpreted genomic data and wrote much of the manuscript. All authors read and approved the final manuscript.

## AVAILABILITY OF DATA AND MATERIALS

Sequence data generated during the current study are available at the Illinois Data Bank (https://databank.illinois.edu/). Seed stocks used during the current study are available from the corresponding author on reasonable request.
