# Supplementary figures and images for: A sorghum NAC gene is associated with variation in biomass properties and yield potential
Source: Plant Direct. 2018 Jul 23;2(7):e00070. doi: 10.1002/pld3.70 (PMC6508854; doi:10.1002/pld3.70)

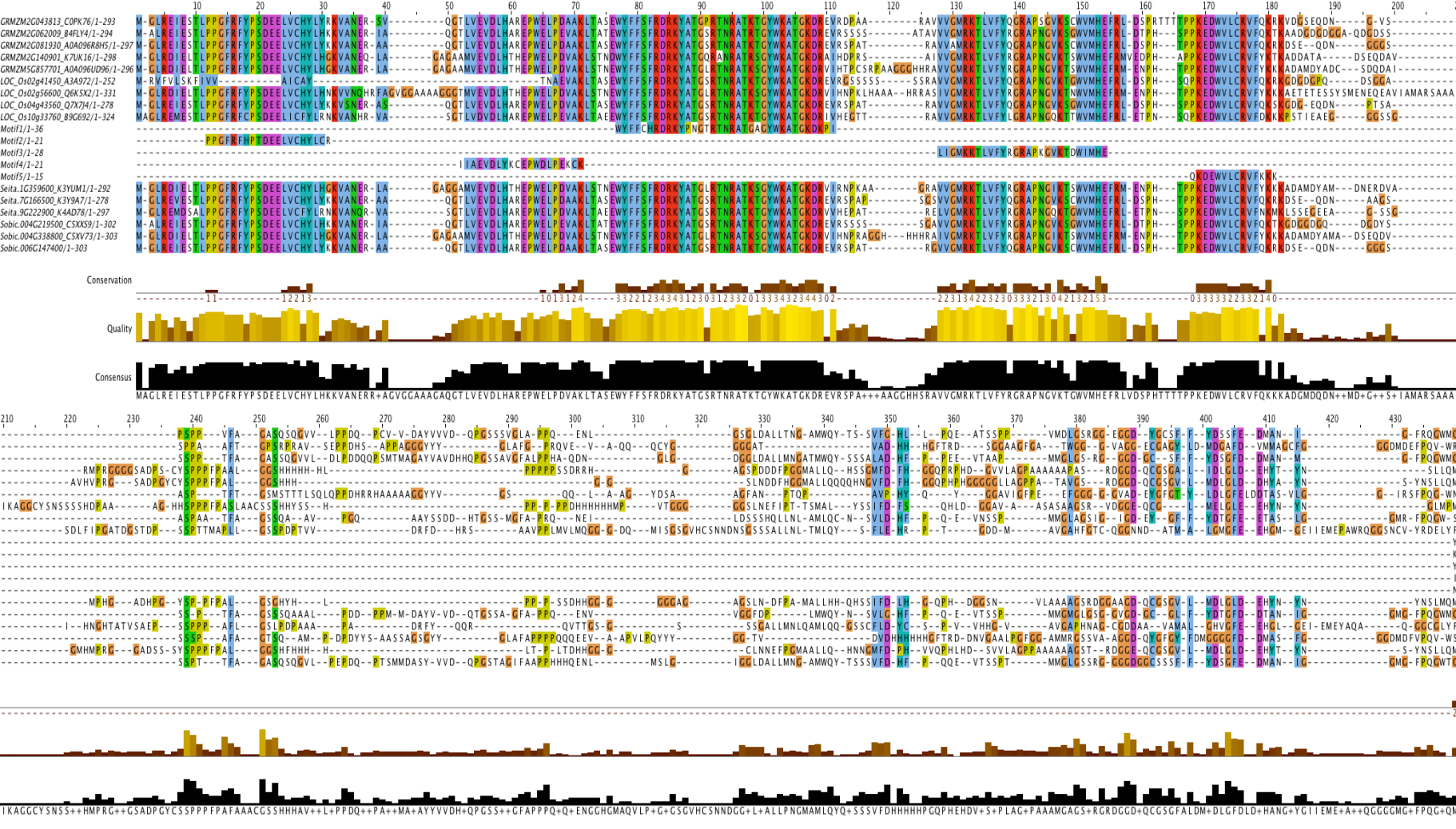

Supplement: Supplementary file 1 [file PLD3-2-e00070-s001.pdf]

## A. Stalk lodging

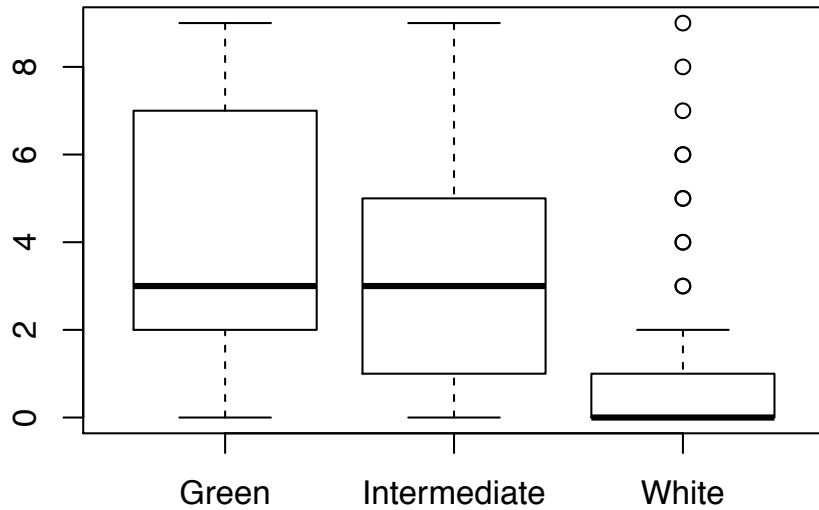

## B. Root lodging

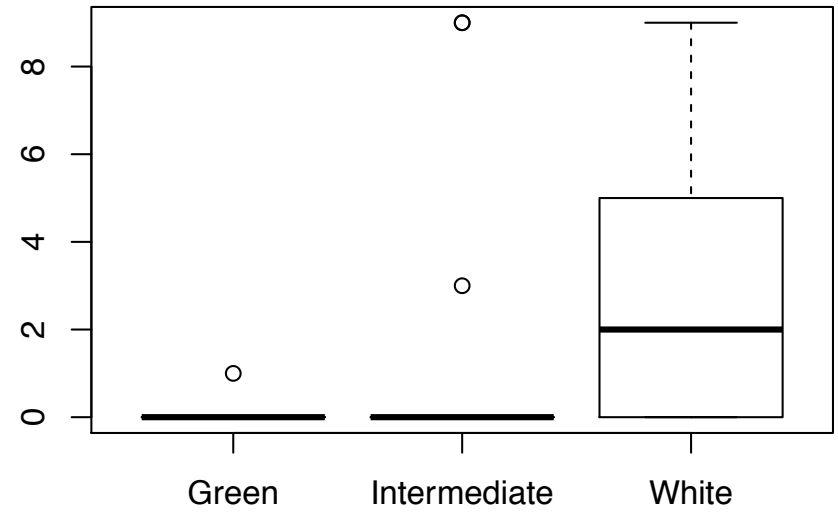

Supplement: Supplementary file 3 [file PLD3-2-e00070-s003.pdf]
