# Supplementary material for: A sorghum NAC gene is associated with variation in biomass properties and yield potential
Source: Plant Direct. 2018 Jul 23;2(7):e00070. doi: 10.1002/pld3.70 (PMC6508854; doi:10.1002/pld3.70)
Supplement: Supplementary file 2 [file PLD3-2-e00070-s002.pdf]

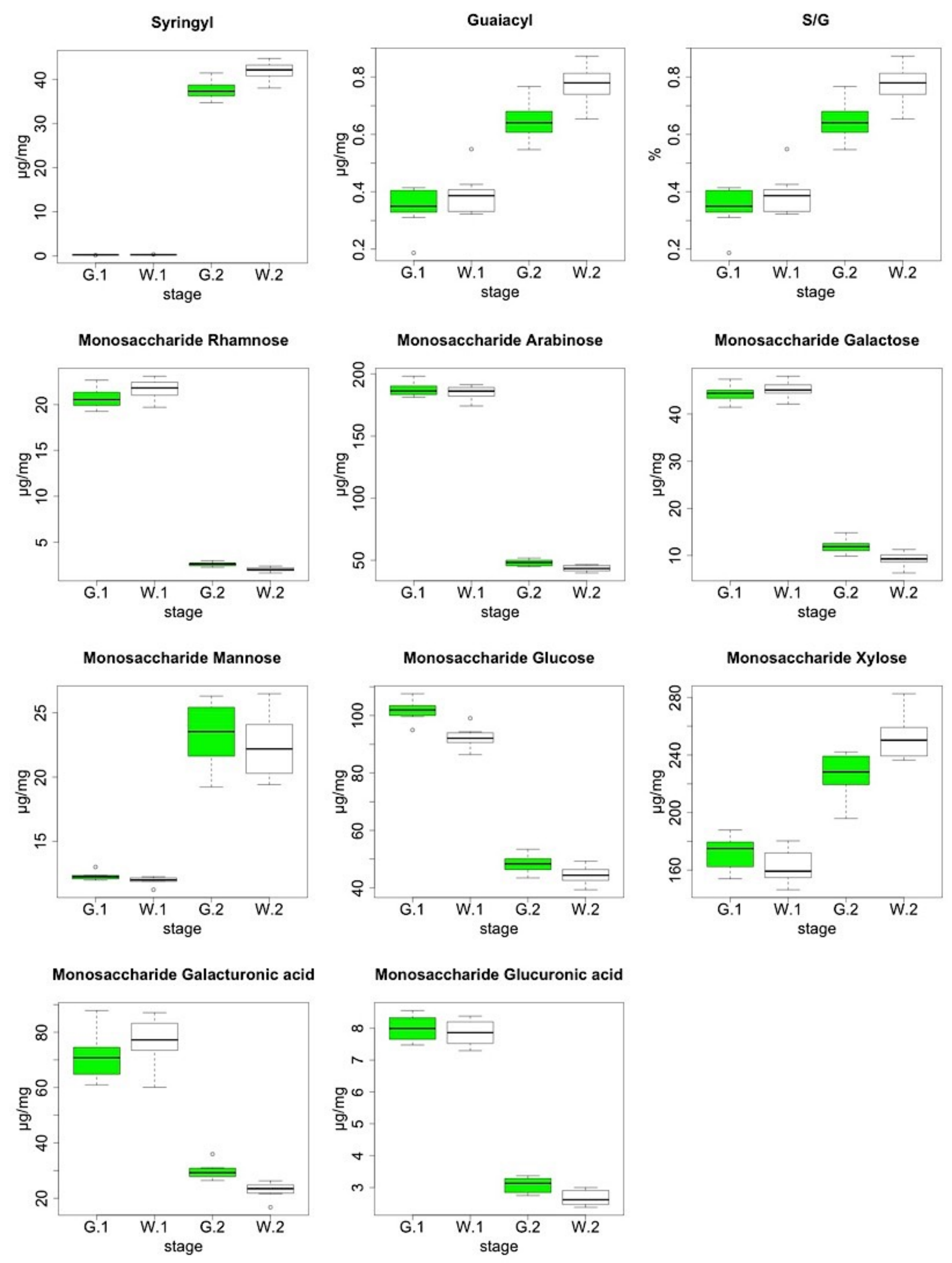
Syringyl
Guaiacyl
S/G
Monosaccharide Rhamnose
Monosaccharide Arabinose
Monosaccharide Galactose
Monosaccharide Mannose
Monosaccharide Glucose
Monosaccharide Xylose
Monosaccharide Galacturonic acid
Monosaccharide Glucuronic acid
μg/mg
%
stage
G.1
W.1
G.2
W.2
