# Supplementary material for: A sorghum NAC gene is associated with variation in biomass properties and yield potential
Source: Plant Direct. 2018 Jul 23;2(7):e00070. doi: 10.1002/pld3.70 (PMC6508854; doi:10.1002/pld3.70)
Supplement: Supplementary file 5 [file PLD3-2-e00070-s005.pdf]

Reviewer 1
Line 127 - Are there any meaningful mutations in Sobic.006G147600? Are there any other mutations in the NAC transcription factor besides the ones listed?
Sobic.006G147600 (threonine aldolase) was not sequenced. Exons and introns of Sobic.006G147400 (NAC) were sequenced, and while the premature stop codon is the most plausible structural mutation, we cannot exclude the possibility that other regulatory mutations affect the function of this NAC gene.

Line 152 - "extracted 1 m of row 30 days after anthesis" Unclear, please revise.
The phrase is revised to "we grew NILs in six replications of paired rows, extracted total sugar from 1 meter (m) of row 30 days after anthesis (see Methods)," (Line 153). As cited in the methods, reference #26 contains more details about phenotyping for total sugar yield.

Line 176 - "marginally significant decrease" - Significant or not? Reported as 0.055, is significance threshold $p < 0.05$?
The phrase is revised to "while their decrease in crystalline cellulose is not significant at $p<0.05$ ($p=0.055$; Figure 6C). (Line 177).

Line 187 - "analysis using WGCNA" - please explain in brief this method.
This phrase was modified to "Co-expression analysis using WGCNA's step by step network construction and module detection" (Line 194). Parameters for network construction and module detection are described in the Methods section (Line 349).

Line 198 - What is the expression of the ZF-HD TF on Chr 1 that was found during GWAS? Is it expressed? Does the expression change? Is there reason to believe it is also implicated in this process?
There is no gene expression for the ZF-HD TF in either dd nor DD. We have added the following sentence at Line 206: "However, this ZF-HD transcription factor (Sobic.001G112500) shows no expression in any of the DD or dd samples.".

Line 286 - Zero principal components were used in the GWAS analysis? Where the same markers found as significant in the model with 1-3 PCs? Was any inclusion of kinship incorporated to account for the population structure within the 1,624 sorghum panel? Is there any information about the panel itself? Any confounding factors in terms of geographic origin, flowering time, etc? What is base number of populations within this panel according to STRUCTURE?
The kinship matrix, but not principal components, was included to control for population structure in this GWAS. The Model.selection=T statement was used in GAPIT to select the optimal number of principal components to include (0-10), and the optimal number (BIC value closest to zero) was zero principal components. However, we note that the association near 51 Mb on chr6 is consistently detected regardless of what model is used to control for population structure, and is even detected if a naïve model (no control for population structure) is used. The panel has not been described previously, but is an expansion of a slightly smaller panel (n=1160)

described by Thurber et al, Genome Biology 2013 14:R68. We have not used STRUCTURE to define populations in this panel.

Line 303, 315, 312, - Revise, line ending in citation. Revise, "according to {citation}".
Revised.

Line 318 - Underscore.
Revised.

Line 324 - Which additional S. bicolor line was used for this analysis? Were the other species mentioned used the references for the respective versions, which version of each genome was used?
The DD NIL was used for this analysis. We revised the sentence to "The full length sequence of SbNAC074a from the DD NIL, which doesn't have the null mutation, was BLASTED in UNIPROT ([http://www.uniprot.org)](http://www.uniprot.org) and homologs from Arabidopsis thaliana (sequence version 1), Oryza sativa subsp. Japonica (sequence version 2 for Os04g43560, others were sequence version 1), Sorghum bicolor (sequence version 1), Zea mays (sequence version 1), Setaria italica (sequence version 1), and Glycine max (sequence version 1) were identified from the BLAST result."(Line344).

Line 489 - "GWAS hits significant at q" q?
q-values, also called FDR-adjusted p-values, are used in the control of the false discovery rate. The q-value represents the minimum FDR at which that test would be considered significant.

Line 502 - 3 Representative examples? Or from three separate plants? Please clarify.
These are representative examples of DD and dd midribs and stem cross-sections at the different growth stages, selected after observing many hundreds of plants.

Line 511 - Is the stop codon the only mutation?
There are three other polymorphisms between the DD and dd alleles of the NAC gene: two synonymous SNPs and a small insertion at the 3' end of the gene.

Figure 6 - which line is which? Fix axis or clarify in caption.
We recreated the Figure and clarified the caption (Line554)

Figure S3 - Missing completely? Please add in figure for review.
Our apologies --we have uploaded this missing figure.

Reviewer2
Reviewer #2:

Xia et al., mapped the sorghum Dry Stalk (D) locus to a small interval with four genes. NAC transcription factor was identified as their candidate. They created NILs and characterized a number of phenotypic traits for these NILs. They also conducted transcriptomic analyses and

GWAS to associate a ZF-HD gene with midrib trait. Sorghum is an important crop for grain and bioenergy. The dissection of the genetic control for stalks and midrib is critical for further crop improvement. The results presented in this paper is solid. I think it would be of interest for the plant direct readers. Below, I have some minor comments.

Minor Comments:
In abstract, lines 43, "dd NILs produce ..." sounds like a simple repeat of lines 34-35.
We deleted "dd NILs produce" in line 43.

In Figure 2 and table S2, the authors presented GWAS results and the corrected p-values, but it would be interesting to report the variance explained by the SNPs, their directions of the effects and the magnitudes of the effects. This is relevant, it will help us to understand what is the contribution of others alleles except for the D locus.

The r2 of a GWAS model including only the K matrix is 0.44. The r2 of a GWAS model including the K matrix and the most significant SNP near 51 Mb on chr56 is 0.49. Therefore the top SNP only explains 5% of additional variance on top of population structure. However, some of the SNP effect is undoubtedly absorbed by the K matrix. Also, we should note that the SNP causing the premature stop codon in Sobic.006G147400 was not included in the GWAS analysis, as we did not genotype this SNP across the entire panel. Therefore the LD between our hypothesized causal SNP and the top SNP in the GWAS analysis is not known, and for this reason we have refrained from going into more detail in our discussion of the magnitude and direction of the SNP effects in GWAS.
